# Analysis of Efficacy and Safety of Modified Transfrontal Puncture Drainage in Hypertensive Basal Ganglia Hemorrhage Patients

**DOI:** 10.3389/fsurg.2022.837008

**Published:** 2022-03-29

**Authors:** Wenxin Wang, Wei Lv, Jianquan Yang

**Affiliations:** ^1^Department of Neurosurgery, Renmin Hospital, Hubei University of Medicine, Shiyan, China; ^2^Department of Neurosurgery, Wuhan Asia General Hospital, Wuhan, China

**Keywords:** modified transfrontal puncture drainage, hypertensive basal ganglia hemorrhage, efficacy, safety, basal ganglia hemorrhage

## Abstract

**Objective:**

The study aimed to explore the efficacy and safety of modified transfrontal puncture drainage in patients with hypertensive basal ganglia hemorrhage.

**Methods:**

The study enrolled 102 patients with hypertensive basal ganglia hemorrhage who received treatment at our hospital between April 2020 and June 2020. They were divided into a control group (51 cases, burr hole evacuation of intracranial hematoma) and a study group (51 cases, modified transfrontal puncture drainage) using the random number table method. The operative time, hematoma evacuation rate, time to recovery of consciousness, postoperative Glasgow coma scales (GOS), and the length of hospital stay were compared between the two groups. The postoperative recovery of neurological function in the two groups was observed, and activities of daily living at 3 months postoperatively in the two groups were statistically analyzed. The postoperative complications and recurrent bleeding, as well as prognosis in the two groups, were recorded.

**Results:**

The operative time, hematoma evacuation rate, time to recovery of consciousness, postoperative GOS scores, time to extubation, and the length of hospital stay of the two groups were compared postoperatively, and the difference was statistically significant (*p* < 0.05). The preoperative neurological function of the two groups was compared, and the difference was statistically insignificant (*P* > 0.05). The postoperative neurological function of the study group was lower than that of the control group, and the difference was statistically significant (*P* < 0.05). The postoperative incidence of stress ulcer, renal failure, and recurrent bleeding in the two groups was compared, and the difference was statistically insignificant (*p* > 0.05). The rate of pulmonary infections and gastrointestinal bleeding in the study group was lower than that of the control group, and the difference was statistically significant (*P* < 0.05). The mortality rate of the study group was 1.96% (1/51) and that of the control group was 3.92% (2/51), and the difference was statistically insignificant (*p* > 0.05). The activities of daily living in the two groups were compared and the difference was statistically insignificant (*p* > 0.05).

**Conclusion:**

Modified transfrontal puncture drainage can effectively treat hypertensive basal ganglia hemorrhage patients and has relatively good safety.

## Introduction

Data indicated that the incidence of cerebral hemorrhage is about 60–80/100,000 persons in China, and the mortality rate of patients with cerebral hemorrhage in the acute stage reached 30–40% (1). Cerebral hemorrhage is a common disease of the nervous system and is mainly associated with cerebrovascular diseases. Meanwhile, hypertensive cerebral hemorrhage is an abrupt brain parenchymal hemorrhage triggered by hypertension, the most common of which is basal ganglia hemorrhage (2). The main features of hypertensive cerebral hemorrhage are the attack rate, severity of the disease, and poor prognosis, and if prompt treatment is not carried out, intracranial hematoma will continue to increase and ICP will continue to rise, which may compress adjacent brain tissues and cause severe brain injury, severely impacting on the normal functioning of the patients and even leading to death (3). Therefore, prompt evacuation of intracranial hematoma, lowering of ICP, and avoiding secondary brain injury are the prime aims of clinical treatment in patients with hypertensive basal ganglia hemorrhage. Clinically, if the volume of hypertensive basal ganglia hemorrhage is small, conservative pharmacotherapy is given and if the volume of cerebral hemorrhage is >30 ml, surgical treatment is required (4). Currently, burr hole intracranial hematoma evacuation and minimally invasive drainage are often used clinically for the surgical treatment of hypertensive basal ganglia hemorrhage. Among them, the operative time of burr hole intracranial hematoma evacuation is long and the prognosis is poor in some patients (2, 5). Modified transfrontal puncture drainage uses local anesthesia based on minimally invasive drainage, and after the selection of puncture sites is done, a puncture needle is advanced into the hematoma and liquified hematoma is partially aspirated and drained to relieve the occupying effect. Meanwhile, in combination with fibrinolytic agents, lytic drainage is undertaken to gradually evacuate the remaining hematoma, thereby lowering ICP (6). Currently, there are only a few clinical reports on the treatment of patients with hypertensive basal ganglia hemorrhage by modified transfrontal puncture drainage. Therefore, the current study aimed to explore the efficacy and safety of modified transfrontal puncture drainage in patients with hypertensive basal ganglia hemorrhage.

## Materials and Methods

### Clinical Materials

#### General Materials

The study enrolled 102 patients with hypertensive basal ganglia hemorrhage who received treatment at our hospital between April 2020 and June 2020. They were divided into the study group and the control group using the random table method, with 51 patients per group. The study group had 30 men and 21 women, and their age ranged between 42 and 76 years with a mean age of (58.98 ± 13.78) years. The control group had 29 men and 22 women, and their age ranged between 43 and 75 years, with a mean age of (58.61 ± 14.04) years. The two groups were comparable in the baseline variables (*P* > 0.05, Supplementary Table 1). The study protocol met the requirements of Helsinki Declaration and was approved by the ethics committee of our hospital.

### Inclusion and Exclusion Criteria

#### Inclusion Criteria

The inclusion criteria were: patients who met the diagnostic criteria of hypertensive cerebral hemorrhage in the basal ganglia, which was confirmed by MRI or CT; initial attack; abnormally elevated blood pressure at admission or a history of hypertension; clear surgical indications; blood loss of 30–60 ml measured by the Tada formula and no bleeding into the cerebral ventricle; a history of hypertension or abnormally elevated blood pressure at admission; receipt of surgical treatment at our hospital; and patients provided written informed consent.

#### Exclusion Criteria

Exclusion criteria were: concomitant hepatic and renal insufficiency; contraindicated for neurosurgery; critically ill at the time of admission; concurrent mental illness or other serious illnesses; concurrent serious somatic diseases or coagulation dysfunction; brain hernia and brain malignancy; preoperative cerebral hernia; cardiac, hepatic, pulmonary, and renal dysfunction; or concurrent serious systemic illness or organ failure.

### Methods

The control group received burr hole intracranial hematoma evacuation and were intubated under general anesthesia. The patient was placed in the supine position with the head tilted to the healthy side and an incision, 4–5 cm in length, was made under the guidance of cerebral CT in the scalp where the hematoma was the largest and the scalp was the most superficial. The scalp and temporalis muscle were incised to fully expose the cranium. The incision was distracted using a mastoid process distractor. After a hole was drilled, the bone window was enlarged to a diameter of 2.5–3.0 cm, and the dura mater was incised in the shape of a cross. Based on CT location data, the hematoma was evacuated using a brain puncture needle and the cortex was incised and dissected until the cavity of the hematoma. The hematoma and clots were aspirated; if there was active punctiform bleeding, hemostasis by sponge compression/electrocoagulation was done. The drainage tube was placed after no punctiform bleeding was present. The incision was closed and sutured, and the drainage tube was removed based on the CT results.

**Figure 1:**
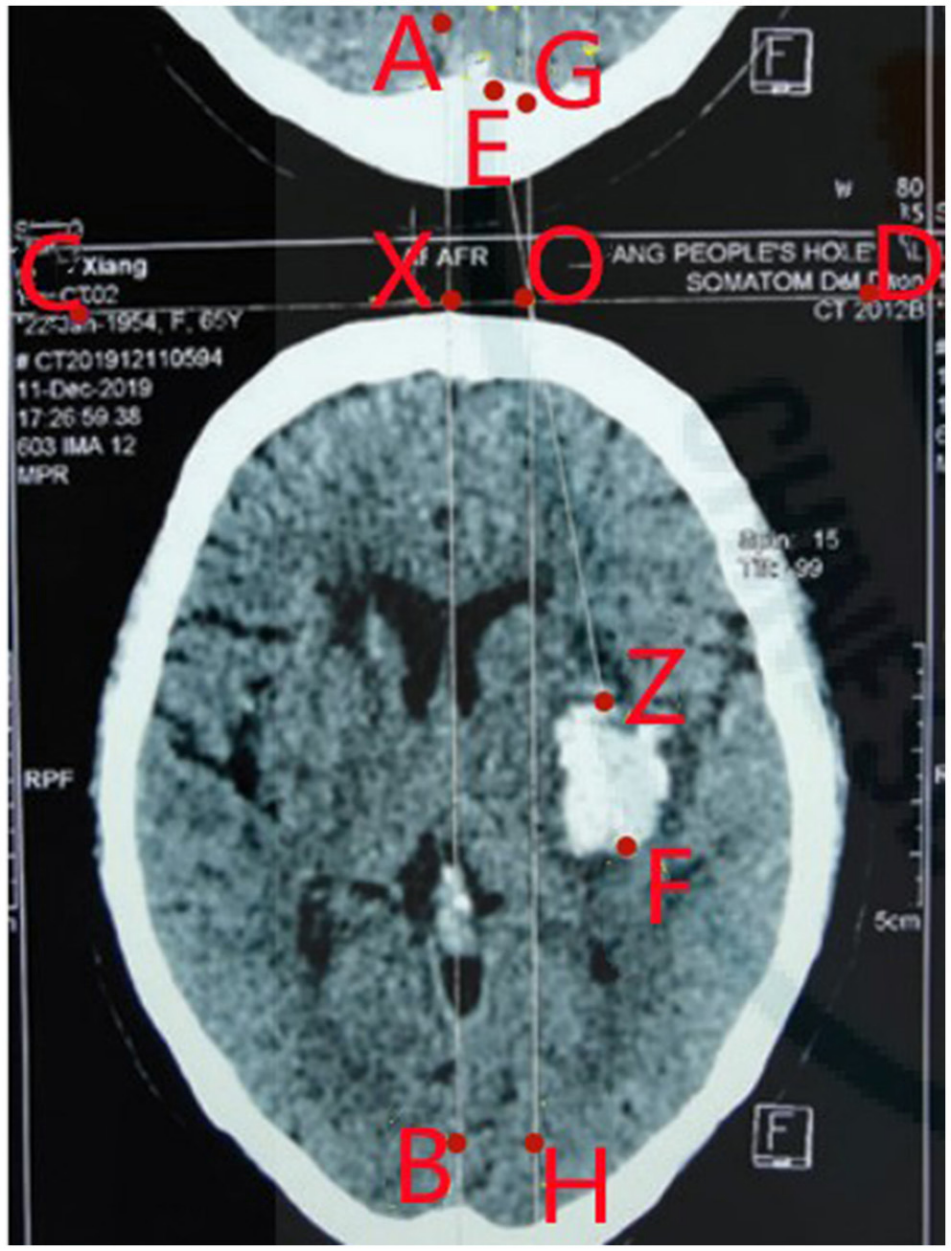
The cerebral CT image. A: A point on the midline (vertical line) of the CT image; B: Another point on the midline of the CT image; C: A point on the vertical line (horizontal line) of the midline AB, the horizontal line is close to the scalp; D: The midline AB A point on the vertical line (horizontal line) of the puncture track; E: a point on the reverse extension of the puncture tract OF; F: The most distal end of the hematoma; G: A point on the vertical line GH, GH is perpendicular to the horizontal line CD, the vertical point is Point O; X: The intersection of the vertical line AB and the horizontal line CD; O: The puncture point, which is a point on the horizontal line CD, and the line segment XO is 1.5 cm; Z: The intersection of the puncture tract OF and the hematoma.

The study group received modified transfrontal puncture drainage. After the scalp was prepared, the maximal axial section of hematoma was selected according to preoperative cerebral CT images (which can be reconstructed, and according to the approximate canthomeatal line, the bilateral lenses and the bilateral tragi were positioned in the same plane, which served as the basal plane). Line AB was the standard midline, line CD was vertical to line AB and flushed against the skin, and point X is the intersection of line AB and line CD. The length of segment XO was 1.5 cm, ray FE passed through O, and F was the most distal point of hematoma. Segment ZF was the largest vertical axis of hematoma, the puncture drainage tube was placed in the direction of FE, and the angle of inclination for puncture was < EOG (generally 10–14°). The puncture site to the most distal point of hematoma cavity was segment OF (generally 9.0–11.5 cm), and through point O was parallel to line AB and was line GH (Figure 1). A 3-mm incision was made in the scalp at the transfrontal vertebrocranial point with a sharp blade after local anesthesia and the skull was drilled through. The left and right arm of a novel disposable stereotaxic apparatus and the scalp formed an arc EF. Overlapping GH, the midpoint (point of origin) of the protractor base aimed at point O, and the dura mater was punctured using a dura mater puncture needle according to the puncture angle aided by the stereotaxic apparatus. A drainage tube containing the guidewire was then passed through the drilled hole and a puncture was done according to the preoperatively measured angle (< EOG) under the guidance of the stereotaxic apparatus. The catheter was slowly advanced and puncture depth was determined preoperatively (the length of segment OF). When the hematoma was reached, the guidewire was removed, the dark liquid component and semi-solidified blood were aspirated, indicating puncture success, and the drainage tube was left in the hematoma. If cerebral hernia occurred preoperatively, 1/3 of the hematoma was aspirated maximally. If resistance was encountered, aspiration should be discontinued.

### Postoperative Management

**Figure 2:**
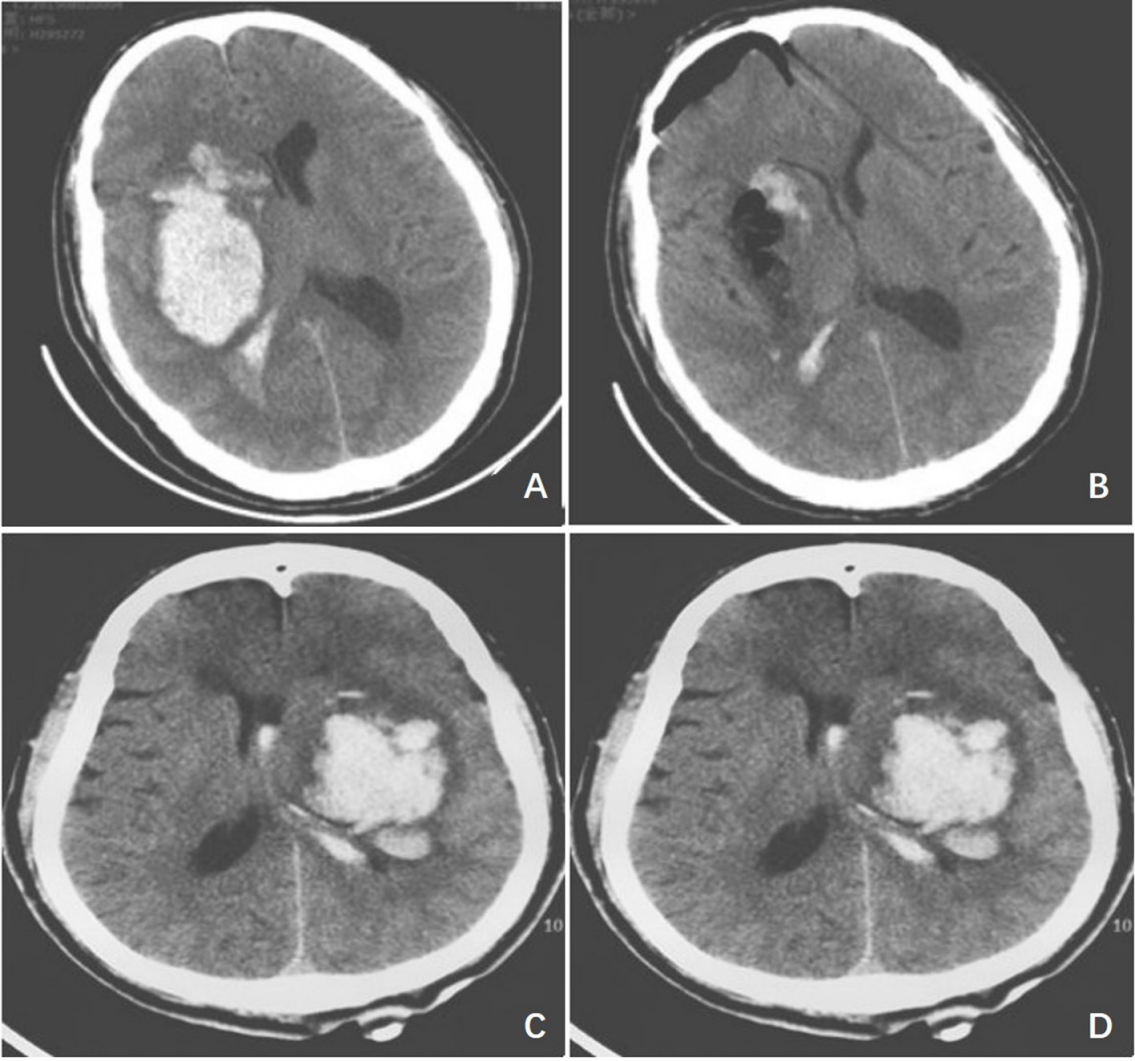
Typical case imaging images. **(A)** The preoperative cerebral CT image of the study group; **(B)** The postoperative cerebral CT image of the study group; **(C)** The preoperative cerebral CT image of the control group; **(D)** The postoperative cerebral CT image of the control group.

Patients were evaluated by postoperative cerebral CT (Figure 2) to observe whether the drainage tube was properly positioned and whether there was bleeding in the puncture tract, and whether bleeding recurred postoperatively. The preoperative and postoperative residual hematoma volume were calculated using the Tada formula and the intraoperative hematoma evacuation rate was also calculated. Intraoperative hematoma evacuation rate = (preoperative hematoma volume - postoperative residual hematoma volume) / preoperative hematoma volume × 100%.

Urokinase (3–100,000 U, 1–2 times/day) was injected daily into the hematoma postoperatively and low drainage was provided. The use of mannitol and other dehydration medications was minimized. Dehydration medications were discontinued in some patients to promote hematoma evacuation. The dose of urokinase was notably reduced in patients with drained hematoma to 50,000 U per injection. Meanwhile, changes in coagulation function were dynamically monitored to avoid urokinase-induced recurrent bleeding of the primary hematoma or puncture tract bleeding. A dynamic cerebral CT scan was done, and the depth of the drainage tube was adjusted promptly based on the scan results. On postoperative day (POD) 3, the severity of coma was evaluated using Glasgow coma scales (GOS). At POD 3 to 6, if the hematoma was completely absorbed and only the drainage tube was seen on cerebral CT, the drainage tube was removed. If hematoma became noticeably smaller and was isodense on cerebral CT scan, drainage was continued. In addition, conventional postoperative management, including active control of ICP, anti-infection, and correction of electrolyte disturbances, was provided.

### Observation Indexes

The operative time, intraoperative blood loss, hematoma evacuation rate, time to recovery of consciousness, postoperative GOS scores, and the length of hospital stay in the two groups were compared. The postoperative recovery of neurological function was observed, and the activities of daily living at 3 months were analyzed. The postoperative complications, recurrent bleeding, and prognosis of the two groups were recorded.

### Neurological Function

Neurological function (7) was evaluated using NISS preoperatively and 1 week postoperatively, with lower scores indicating better neurological function.

A GOS score (8) of five denotes resumption of normal life with minor deficits; four, moderate disability, patients independent in daily life, work under protection; three, severe disability, patient dependent on daily support; two, neurovegetative state, minimal response (eyes can open with asleep/awake cycle); and one, death.

The rates of pulmonary infections, GI bleeding, stress ulcer, renal failure, and recurrent bleeding in the two groups were analyzed.

Activities of daily living at 3 months were evaluated using the Barthel index (BL) (9). Very severe functional impairment is denoted by 0–25; severe functional impairment, 25–50; moderate functional impairment, 50–75; mild functional impairment and basically independent, 75–100; and independent, 100.

### Statistical Methods

Data were analyzed using SPSS 21.0 and quantitative data were expressed in $\left[\bar{x}\pm s\right]$ and compared by the *t*-test. Categorical data were expressed as rate (%) and compared using the chi-square (χ^2^) test. *P* < 0.05 indicated that the difference was statistically significant.

## Results

### Comparison of Operative Time, Hematoma Evacuation Rate, Time to Recovery of Consciousness, Postoperative GOS Scores, Time to Extubation, and Length of Hospital Stay in the Two Groups

Postoperatively, the operative time, time to recovery of consciousness, time to extubation, and the length of hospital stay were shorter in the study group than those of the control group. There was statistically a significant difference in the hematoma evacuation rate and postoperative GOS scores in the two groups (*P* < 0.05), as shown in Table 1.

**Table 1:** Comparison of operative time, hematoma evacuation rate, time to recovery of consciousness, postoperative GOS scores, time to extubation, and length of hospital stay in the two groups, *n* (%).

**Groups**	***n***	**Operative time (min)**	**Hematoma evacuation rate (%)**	**Time to recovery of consciousness (min)**	**Postoperative GOS scores**	**Time to extubation (d)**	**Length of hospital stay (d)**
The study group	51	30.47 ± 3.29	89.98 ± 12.76	5.13 ± 1.04	4.98 ± 1.21	3.04 ± 1.02	11.58 ± 2.09
The control group	51	101.29 ± 15.98	80.32 ± 12.69	8.98 ± 1.12	3.12 ± 0.24	3.72 ± 1.12	14.67 ± 2.11
t/χ^2^		−30.999	3.833	−17.989	10.768	−3.206	−7.430
*P*		<0.001	<0.001	<0.001	<0.001	0.002	<0.001

### Comparison of Recovery of Neurological Function in the Two Groups

There was no statistically significant difference in preoperative neurological function between the two groups (*P* > 0.05). The postoperative neurological function was lower in the study group than the control group, and the difference was statistically significant (*P* < 0.05), as shown in Table 2.

**Table 2:** Comparison of recovery of neurological function in the two groups $\left(\bar{x}\pm s\right)$.

**Groups**	***n***	**Preoperative**	**1 week postoperatively**
The study group	51	18.99 ± 3.01	6.79 ± 1.02
The control group	51	18.89 ± 3.21	10.03 ± 1.23
T		0.162	−14.480
*P*		0.872	<0.001

### Comparison of Postoperative Complications and Recurrent Bleeding in the Two Groups

There was no statistically significant difference in the postoperative rates of stress ulcer, renal failure, and recurrent bleeding between the two groups (*P* > 0.05). The study group had a lower rate of pulmonary infections and GI bleeding than the control group, and the difference was statistically significant (*P* < 0.05), as shown in Table 3.

**Table 3:** Comparison of postoperative complications and recurrent bleeding in the two groups, *n* (%).

**Groups**	***n***	**Pulmonary infections**	**GI bleeding**	**Stress ulcer**	**Renal failure**	**Recurrent bleeding**
The study group	51	1 (1.96)	1 (1.96)	3 (5.88)	2 (3.92)	3 (5.88)
The control group	51	8 (15.69)	8 (15.69)	4 (7.84)	3 (5.88)	4 (7.84)
T		5.971	5.971	0.153	0.210	0.153
*P*		0.015	0.015	0.695	0.647	0.695

### Prognosis

The mortality rate of the study group was 1.96% (1/51) and that of the control group was 3.92% (2/51), and the difference was statistically insignificant (*P* > 0.05). There was no statistically significant difference in the activities of daily living between the two groups (*P* > 0.05), as shown in Table 4.

**Table 4:** Comparison of activities of daily living in the two groups, *n* (%).

**Groups**	***n***	**Very severe functional impairment**	**Severe functional impairment**	**Moderate functional impairment**	**Mild functional impairment**	**Independent**
The study group	51	1 (1.96)	1 (1.96)	1 (1.96)	3 (5.88)	45 (88.24)
The control group	51	2 (3.92)	2 (1.96)	4 (7.84)	2 (5.88)	41 (80.39)
T		0.343	0.343	1.893	0.210	1.186
*P*		0.558	0.558	0.169	0.647	0.276

## Discussion

Cerebral hemorrhage is caused by non-traumatic rupture of the brain parenchymal vessels and accounts for 75% of cerebral hemorrhage cases. Basal ganglia hemorrhage accounts for up to 60% of all types of cerebral hemorrhage (10). The hypertensive cerebral hemorrhage causes a persistent rise in ICP due to the occupying effect of intracranial hematoma, leading to brain tissue edema and secondary brain ischemia and further expanding the extent of compression of adjacent brain tissues and aggravating brain edema, thus forming a vicious cycle (11). This also accounts for the high morbidity and mortality rate of patients with hypertensive cerebral hemorrhage. Previous studies have shown that because hypertensive basal ganglia hemorrhage requires prompt surgical treatment, though the surgical field of conventional craniotomy and craniectomy is clear and can completely evacuate hematoma and promptly lower ICP, the surgery is invasive, has a large volume of blood loss, and patients need a long course of postoperative recovery and have many complications with a poor prognosis (12). With continuing advances in medical technology, microscopic techniques have been widely used in clinical treatment, and burr hole intracranial hematoma evacuation and minimally invasive burr hole drainage have been promoted clinically. Burr hole intracranial hematoma evacuation is performed under direct vision and, on being assisted by a microscope, it can achieve relatively complete evacuation hematoma and eliminate punctiform bleeding. Compared with conventional craniotomy, the surgical incision, bone window area, and the extent of brain tissue exposure are smaller, thus lessening surgical trauma (13). However, burr hole intracranial hematoma evacuation requires tracheal intubation under general anesthesia, separation of brain parenchyma, and has a high demand of surgical skills. It also has a longer operative time and may damage brain tissues (13). Meanwhile, modified transfrontal puncture drainage does not require craniotomy and is undertaken assisted by the brain stereotaxic apparatus under nerve navigation and can precisely puncture intracerebral hematoma and aspirate the hematoma, promptly relieving the occupying effect. For non-evacuated hematoma, postoperative lysis, by multiple injections of urokinase and fibrinolytic agents, and drainage are done to gradually evacuate residual hematoma, thereby relieving the clinical symptoms of the patients.

The results of the current study indicated that the operative time, hematoma evacuation rate, time to recovery of consciousness, postoperative GOS scores, time to extubation, and length of hospital stay were significantly different between the two groups (*P* < 0.05), suggesting that modified transfrontal puncture drainage could effectively shorten the operative time, time to recovery of consciousness, time to extubation, and length of hospital stay of patients with hypertensive basal ganglia hemorrhage. This is probably because the basal ganglia is mainly supplied by the lenticulostriate arteries from the M1 segment of the middle cerebral artery. Punctiform bleeding usually occurs inferior to the hematoma, and transcranial puncture may injure the veins, arteries, and cisterns at the lateral fissure while transfrontal puncture of hematoma could effectively avoid disturbing punctiform bleeding as it avoids the surrounding vessels lateral to the lateral fissure, though the puncture path is longer than that of transcranial puncture, and it punctures the hematoma anteroposteriorly along the long axis of the hematoma, thereby reducing the rate of recurrent bleeding due to suction and drainage. In addition, patients are mainly placed supine, and when the puncture is done from the forehead, the distal portion of the drainage tube lies in the distal part of the hematoma. When hematoma gradually becomes liquified, it flows out of the side holes of the proximal drainage tube. When the most posterior hematoma has disappeared, the drainage tube can be sightly pulled out to reduce the length of the indwelling drainage tube in the brain to aspirate the residual hematoma. Moreover, puncture along the long axis of the hematoma facilitates the injection of urokinase *via* the drainage tube into the hematoma cavity to fully contact the hematoma and expedite hematoma dissolution, thus shortening drainage duration and reducing the risk of postoperative infections. Meanwhile, transcranial puncture cannot achieve the above effects.

Shi *et al*. (14) divided 56 basal ganglia hemorrhage patients into two groups, the control group that received hematoma drainage *via* cranial puncture and the observation group that underwent transfrontal hematoma puncture drainage, and the results showed that transfrontal hematoma puncture drainage had notable effects in the treatment of basal ganglia hemorrhage, leading to symptomatic improvement, lowering hematoma volume and the rate of complications, and increasing the rate of hematoma evacuation. Dong *et al*. (15) studied 124 patients with hypertensive basal ganglia hemorrhage and divided the patients into the craniectomy group (72 patients) and the puncture drainage group (52 patients), and the results showed that transfrontal stereotactic puncture drainage for the treatment of basal ganglia hypertensive cerebral hemorrhage may shorten the operative time, reduce the intraoperative volume of blood loss, effectively promote the recovery of neurological function and activities of daily living of the patients, and lower the rate of postoperative complications. The results of the current study showed that the postoperative neurological function of the study group was lower than that of the control group (*P* < 0.05), suggesting that modified transfrontal puncture drainage could effectively improve the neurological function of patients with hypertensive basal ganglia hemorrhage. The reasons are as follows: (1) the puncture plane was determined by preoperative cerebral CT images in modified transfrontal puncture drainage, and the puncture plane and puncture site are marked in the scalp of the patients. The puncture site and depth are determined by preoperative cerebral CT images, which can effectively increase the precision of puncture and avoid injury to the cortex of the spinal cord and vessels. (2) In modified transfrontal puncture drainage, the operative time is shorter and can rapidly evacuate hematoma early, relieving the compression of brain tissues by hematoma, lessening and even blocking the occurrences of brain edema, effectively preventing a persistent rise in ICP, and lessening or blocking secondary brain tissue injury, thereby improving the neurological function of patients.

Guo *et al*. (16) divided 90 patients with basal ganglia hemorrhage into the control group and the observation group using the random table method, with 45 cases per group. The control group received craniotomy hematoma evacuation, and the observation group underwent stereotactic transfrontal puncture drainage. The results showed that transfrontal stereotactic puncture drainage may lessen the inflammatory reaction of the patients with basal ganglia hemorrhage and lower the severity of damage of neurological function. Hence it is recommended for clinical use. Guan *et al*. (17) carried out a prospective controlled study of 68 hypertensive basal ganglia hemorrhage patients without cerebral hernia; 24 patients received transfrontal stereotactic puncture drainage (the puncture group) and 44 cases underwent hematoma evacuation (the craniotomy group). The results showed that transfrontal stereotactic puncture drainage may be the first choice for patients with hypertensive basal ganglia hemorrhage without a cerebral hernia, and it can lower the rate of pulmonary infections and other complications. Craniotomy hematoma evacuation can be used as a rescue surgery for patients who had postoperative recurrent bleeding. The results of the current study showed that the rates of postoperative pulmonary infections and GI bleeding were lower in the study group than those of the control group (*P* < 0.05), suggesting that modified transfrontal puncture drainage could effectively lower the rates of pulmonary infections and GI bleeding in patients with hypertensive basal ganglia hemorrhage with better safety. This is likely because burr hole intracranial hematoma evacuation belongs to craniotomy and has poor tightness; therefore, there is the possibility of infections. In addition, the procedure is performed under general anesthesia and aspiration could occur during tracheal extubation, increasing the risk of pulmonary infections. Furthermore, the procedure requires separation of the brain parenchyma and has a high demand for surgical skills. The brain tissues and cells may be injured during traction, aggravating postoperative stress, leading to a poor postoperative prognosis and increasing the rate of GI bleeding. Modified transfrontal puncture drainage does not require craniotomy for evacuation hematoma, is easy to perform, is less time-consuming, less invasive, has better tightness, and effectively avoids postoperative infections and other craniotomy-related complications. In addition, combined with urokinase lysis and aspiration drainage, it does not require separation and traction of the brain parenchyma and can better evacuate hematoma in deep brain tissues, relieving occupying effects. Therefore, the procedure causes less injury to the surrounding vessels and nerves with smaller stress, with better safety.

Wang *et al*. (8) studied 72 patients with hypertensive basal ganglia hemorrhage who were treated with transfrontal puncture drainage, and the results showed that transfrontal puncture drainage in these patients was less invasive, had satisfactory efficacy, and could improve the activities of daily living of the patients. The results of the current study demonstrated no statistical difference in the morbidity and mortality rate and activities of daily living between the two groups (*P* > 0.05), suggesting that modified transfrontal puncture drainage could have a similar survival rate to that of burr hole intracranial hematoma evacuation and comparable activities of daily living. This is likely because modified transfrontal puncture drainage may improve the prognosis of hypertensive patients with basal ganglia hemorrhage and raise the activities of daily living. The procedure can rapidly evacuate hematoma, achieving hemostasis. Meanwhile, it avoids neurotoxicity of the hematoma degradation products. The incision is small and generates smaller compression of brain tissues and cells. Furthermore, hemorrhage can be monitored by CT, effectively preventing recurrent bleeding, thereby increasing the activities of daily living.

There are still limitations in our study. First, the small sample size may have limited our ability to identify a statistically significant difference in pre- and postoperative; second, all data were not collected by multiple surgeons, which may have a certain degree of bias. In addition, the postoperative follow-up time of the patients was short, only 3 months. But our research is not stagnant, and it is believed that in the following studies, more patients will be included, the follow-up will be longer, and the results will be more convincing.

In summary, modified transfrontal puncture drainage can shorten the operative time, time to recovery of consciousness, time to extubation, and length of hospital stay of patients with hypertensive basal ganglia hemorrhage. It also can improve neurological function, reduce the rate of pulmonary infections and GI bleeding, and increase the activities of daily living, thereby improving the prognosis of these patients. However, the procedure is performed under a nerve navigator and a stereotactic apparatus, which are complex and expensive devices that is difficult to be promoted in the primary care setting. In addition, drill drift may occur when an electric drill is used during the operation and the puncture plane may shift as a result of protractor board shift when the stereotaxic apparatus is installed, requiring further improvement.

## Data Availability Statement

The original contributions presented in the study are included in the article/Supplementary Material, further inquiries can be directed to the corresponding author/s.

## Ethics Statement

The studies involving human participants were reviewed and approved by Renmin Hospital, Hubei University of Medicine. The patients/participants provided their written informed consent to participate in this study.

## Author Contributions

WW and WL contributed to the conception and design of the study. WW and JY performed the experiments and collected and analyzed data. WW, WL, and JY wrote the manuscript and revised the manuscript. All authors reviewed and approved the final version of the manuscript.

## Conflict of Interest

The authors declare that the research was conducted in the absence of any commercial or financial relationships that could be construed as a potential conflict of interest.

## Publisher's Note

All claims expressed in this article are solely those of the authors and do not necessarily represent those of their affiliated organizations, or those of the publisher, the editors and the reviewers. Any product that may be evaluated in this article, or claim that may be made by its manufacturer, is not guaranteed or endorsed by the publisher.

## Abbreviations

BL: Barthel index
POD: postoperative day.
