# Supplementary material for: Analysis of Efficacy and Safety of Modified Transfrontal Puncture Drainage in Hypertensive Basal Ganglia Hemorrhage Patients
Source: Front Surg. 2022 Mar 29;9:837008. doi: 10.3389/fsurg.2022.837008 (PMC9001930; doi:10.3389/fsurg.2022.837008)
Supplement: Supplementary file 1 [file Table_1.DOCX]

Supplementary Table 1. Comparison of baseline variables between the two groups

| Variables | The study group (n=51) | The control group (n=51) | t/χ^2^ | P |
| --- | --- | --- | --- | --- |
| Sex (M/F, n) | 30/21 | 29/22 | 0.040 | 0.841 |
| Age (years) | 58.98±13.78 | 58.61±14.04 | 0.134 | 0.894 |
| Smoking (n) | 27 | 25 | 0.157 | 0.692 |
| Drinking (n) | 26 | 23 | 0.353 | 0.552 |
| Education (n) |  |  |  |  |
| Elementary school and below | 12 | 10 | 0.232 | 0.630 |
| Middle school | 15 | 13 | 0.197 | 0.657 |
| High school/specialty school | 14 | 16 | 0.189 | 0.664 |
| Polytechnic | 7 | 8 | 0.078 | 0.780 |
| College and above | 3 | 4 | 0.153 | 0.695 |
| Receipt of surgery within 6 hours of hemorrhage | 12 | 11 | 0.056 | 0.813 |
| Receipt of surgery within 6-24 hours of hemorrhage | 35 | 37 | 0.189 | 0.664 |
| Receipt of surgery within 1-3 days of hemorrhage | 4 | 3 | 0.153 | 0.695 |
| Estimated blood loss (mL) | 48.38±4.39 | 48.29±4.41 | 0.103 | 0.918 |
| Preoperative Glascow Coma Scale (GOS) | 8.47±1.87 | 8.41±1.83 | 0.164 | 0.870 |
| Location of hemorrhage (n) |  |  |  |  |
| Left | 27 | 24 | 0.353 | 0.552 |
| Right | 26 | 25 | 0.039 | 0.843 |
| Putamen | 31 | 29 | 0.991 | 0.319 |
| Internal capsule | 13 | 15 | 0.197 | 0.657 |
| Others | 6 | 7 | 0.088 | 0.767 |
| Course of hypertension (years) | 19.73±3.29 | 19.67±3.32 | 0.092 | 0.927 |
